# *Hirudo* (Leech) for proliferative vitreous retinopathy: A protocol for systemic review and meta-analysis

**DOI:** 10.1097/MD.0000000000024412

**Published:** 2021-01-22

**Authors:** Hui Huang, Ruxue Lei, Yuanyuan Li, Qun Huang, Na Gao, Weiwen Zou

**Affiliations:** a Hospital of Chengdu University of Traditional Chinese Medicine; b Chengdu University of Traditional Chinese Medicine, Chengdu, Sichuan; c Xi’an Hospital of Traditional Chinese Medicine, Xi’an, Shanxi Province, P.R. China.

**Keywords:** *Hirudo*, leech, meta-analysis, proliferative vitreous retinopathy, protocol, systematic review

## Abstract

**Introduction:**

Proliferative vitreous retinopathy (PVR) is characterized by proliferation of cells and contraction of membranes on either the retinal surface or in the vitreous cavity, which leads to retinal detachment and visual impairment. PVR is commonly seen in patients with rhegmatogenous retinal detachment and diabetic retinopathy, which seriously affects the patient's work and life. Previous studies indicated that *Hirudo* (Leech) or compound prescription containing *Hirudo* (Leech) for treatment of PVR would be effective. However, due to the lack of evidence, there are no specific methods or suggestions, so it is necessary to carry out systematic evaluations on *Hirudo* (Leech) for PVR and provide effective evidence for further research.

**Methods and analysis:**

The following 8 databases will be searched: Cochrane Central Register of Controlled Trials, PubMed, MEDLINE, EMBASE, China National Knowledge Infrastructure, Chinese Biomedical Literature Database, VIP Database, and Wanfang Database. All randomized controlled trials in English or Chinese related to *Hirudo* (Leech) for PVR will be included. Outcomes will include change in Vitreous opacity, Vision changes, production of the anterior macular membrane, and retinal detachment again. The incidence of adverse events will be assessed for safety evaluation. Study inclusion, data extraction and quality assessment will be performed independently by 2 reviewers. Assessment of risk of bias and data synthesis will be performed using Review Manager V.5.3.

**Results:**

In this systematic review and meta-analysis, we will synthesize the studies to assess the safety and efficacy of *Hirudo* (Leech) for PVR.

**Conclusion:**

The summary of our study will clarify whether *Hirudo* (Leech) therapy could be an efficient and safe method for PVR, which can further guide the promotion and application of it.

**Open Science Framework (OSF) registration number:**

10.17605/OSF.IO/FP7VG (https://osf.io/fp7vg)

## 1 Introduction

PVR is characterized by pre-, sub-, or intra-retinal fibrosis (scarring) that can result in recurrent detachments, which is also a condition that arises in 5% to 10% of rhegmatogenous retinal detachment (RRD) and is the leading cause of RD surgery failure.^[1,2]^ PVR with recurrent retinal detachments requires additional surgical interventions and is associated with poor visual outcomes.^[3]^ There are currently no treatments for PVR other than surgery to remove the PVR membranes or excise portions of the retina. Although tremendous progress has been made in the equipment and operation skills in recent years, the surgical treatment is not ideal and cannot avoid recurrence, since vitrectomy itself is one of the common causes of PVR.^[4]^ Studies show that pharmaceutical agents that inhibit PVR development during the retinal detachment repair process could potentially improve both the surgical success rate and visual outcome.^[5,6]^

In recent years, traditional Chinese medicine (TCM) has been widely used in clinical and experimental study of PVR, which has been proven to be fully effective. *Hirudo* (Leech) or compound prescriptions containing *Hirudo* (Leech) are insect-like drugs commonly used in TCM. The exploration of treatment methods that invigorate the circulation of blood show that *Hirudo* (Leech) was strongly effective at improving microcirculation and vitreous hemoptysis,^[7,8,9]^ but research about its effectiveness and safety have not yet reached a definitive conclusion. Consequently, this research intends to adopt a system valuation and meta-analysis method of *Hirudo* (Leech) or compound prescription containing *Hirudo* (Leech) in the treatment of PVR to evaluate its efficacy and safety.

## 2 Methods

### 2.1 Study registration

This protocol of this study has been registered in OSF (Open Science Framework) Preregistration. December 01, 2020. Registration DOI:10.17605/OSF.IO/FP7VG (https://osf.io/fp7vg). The protocol will be conducted severely under the guideline of Preferred Reporting Items for Systematic Reviews and Meta-analyses Protocols.^[10]^ If amendments are needed, the authors will update their protocol to include any changes in the whole process of research.

### 2.2 Include criteria

#### 2.2.1 Type of study

##### 2.2.1.1 Type of participants

1). The patients, aged 18 years or older, suffering from PVR will be included, regardless of the limitation of gender and nationality.
2). Patients in PVR after RRD repair surgery or with diabetic retinopathy also included. Degree of vitreous opacity are not restricted.

##### 2.2.1.2 Type of intervention

The TCM *Hirudo* (Leech) or compound prescription contain *Hirudo* (Leech) should be the main treatments.

Control interventions including studies in which the effects of PVR was compared with no treatment/waiting list, sham control or active treatment (e.g., other ophthalmic surgery, injection, or other traditional medical treatments). Studies in which the effects of PVR were compared with other TCM therapy will be excluded. In case the participants of the PVR group received another active treatment, only studies in which the participants of all comparison groups received the same active treatment as a cointervention will be included.

### 2.3 Exclusion criteria

1). Participants were diagnosed with the unclear diagnostic criteria.
2). Duplicated data or the data cannot be extracted.
3). Non- randomized controlled trials and Quasi- randomized controlled trials.
4). Observational studies and retrospective studies.
5). Animal studies.

### 2.4 Search methods for identification of studies

#### 2.4.1 Electronic data sources

Eight data bases will be searched to identify eligible studies: Cochrane Central Register of Controlled Trials, PubMed, MEDLINE, EMBASE, China National Knowledge Infrastructure, Chinese Biomedical Literature Database, VIP Database, and Wanfang Database. The time range is the starting time is determined according to the first literature available, and the deadline is November 2020.

#### 2.4.2 Other resources

Other resources of related studies will be searched. The PROSPERO Register of Controlled Trials, the Cochrane Central Register of Controlled Trials, and the Cochrane Complementary Medicine Field Specialized Register were also retrieved. Relevant conference papers or other relevant literatures were also conducted. If it is necessary, we will contact with trail author to obtain the latest clinical data.

#### 2.4.3 Search strategy

The following search terms will be used: *Hirudo*/Leech, *Whitmania pigra Whitman*, proliferative vitreous retinopathy/PVR, rhegmatogenous retinal detachment/RRD, diabetic retinopathy/RD, traditional Chinese medicine/TCM, randomized controlled trial/RCT. Different retrieval strategies in Chinese and foreign databases will be used. Language restrictions are Chinese and English. There is no publication restriction. Here we take the search strategy in PubMed as an example and list in Table 1. Additionally, we will make appropriate modifications in accordance with the actual requirements.

**Table 1:** Search strategy sample of PubMed.

Number	Searchs
#1	Proliferative Vitreous Retinopathy (MeSh)
#2	Proliferative Vitreous Retinopathy (ti, ab)
#3	Proliferative Vitreoretinopathy (MeSh)
#4	PVR (ti, ab)
#5	or#1–4
#6	Medicine, Chinese Traditional (MeSh)
#7	Traditional Chinese Medicine (ti, ab)
#8	TCM (ti, ab)
#9	or#6–8
#10	*Hirudo* (MeSh)
#11	*Hirudo* (ti, ab)
#12	Leech (ti, ab)
#13	*Hirudin* (ti, ab)
#14	*Whitmania pigra Whitman* (ti, ab)
#15	or#10-14
#16	Rhegmatogenous Retinal Detachment (MeSh)
#17	RDD (ti, ab)
#18	Retinal Detachment (ti, ab)
#19	Diabetic Retinopathy (MeSh)
#20	DR (ti, ab)
#21	#5 and #16-20
#22	Randomized Controlled Trial (MeSh)
#23	Randomized Controlled Trial (ti, ab)
#24	RCT (ti, ab)
#25	#5 and #9 and #15 and #21 and #25

### 2.5 Types of outcome measures

#### 2.5.1 Primary outcome measures

1). Change in Vitreous opacity.
2). The best corrected visual acuity.
3). Severe adverse events related to the treatment.

#### 2.5.2 Secondary outcome measures

1). Production of the anterior macular membrane.
2). Retinal detachment again.
3). Adverse events related to PVR or any other treatments.

### 2.6 Data extraction

#### 2.6.1 Selection of studies

**Figure 1:**
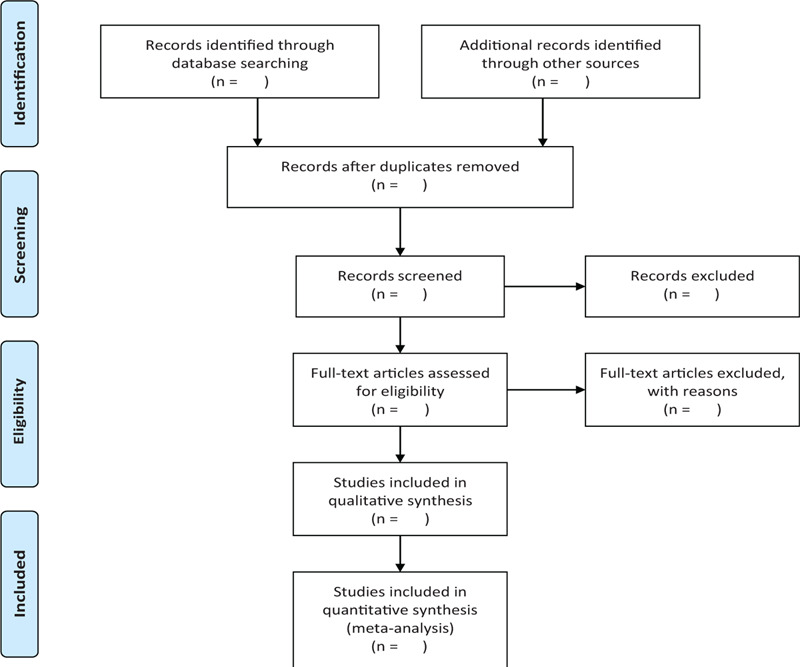
The research flowchart. This figure shows the identification, screening, eligibility, and included when we searching articles.

Two researchers (HH, RXL) will independently obtain the studies from the databases mentioned earlier and access the titles and abstracts of each study, and then exclude the obviously unqualified literature. Later, they will strictly screen the studies by following the eligibility criteria and exclusion criteria. The different opinions will be resolved by discussions. The final selection procedure is indicated in Figure 1 abide by the Preferred Reporting Items for Systematic Reviews and Meta-analyses Protocols guidelines.

#### 2.6.2 Data extraction and management

Retrievals were actualized and data extracted by 2 independent investigators (QH and NG). Each study was evaluated for design, participants’ characteristics, interventions, eligibility criteria, outcomes measures, and research quality, and detail recorded in an Excel file.

#### 2.6.3 Assessment of risk of bias in included studies

The 2 reviewers (HH, RXL) will independently use the bias tool of Cochrane Handbook for Systematic Reviews of Interventions^[11]^ to evaluate the risk of bias of the final included studies. Assessing the risk of bias:

1). random sequence generation;
2). allocation concealment;
3). blinding of participants and personnel;
4). blinding of outcome assessment;
5). incomplete outcome data;
6). selective outcome reporting;
7). other bias.

The quality of the studies will be divided into 3 levels: “low risk of bias,” “high risk of bias,” and “unclear risk of bias.”

#### 2.6.4 Measures of treatment effect

The dichotomous outcomes will be expressed by the odds ratio, while the continuous data will use the standardized mean difference. The 95% confidence interval will be presented for both dichotomous outcomes and continuous outcomes.

#### 2.6.5 Management of missing data

We will take the method of contacting corresponding authors to obtain the missing data. The incomplete data will be dislodged if it cannot be supplement.

#### 2.6.6 Assessment of heterogeneity

Heterogeneity will be assessed by the Cochran Q statistic and quantified by the I^2^ statistic. If I^2^ > 50%, the studies were considered to be heterogeneity, a random-effects models would be used. If I^2^ < 50%, a fixed-effects models were implemented. I^2^ (25%–50%) as moderate level heterogeneity.

#### 2.6.7 Assessment of reporting biases

The bias of publication will be explored through funnel plot analysis. If the funnel plot show asymmetry, it will be evaluated via the Egger and Begg tests, and *P* value < .05 means the publication bias is significant.

#### 2.6.8 Subgroup analysis

When the heterogeneity test results are heterogeneous, we need to clarify the source of the heterogeneity by subgroup analysis. The effects of different types of therapy including design scheme, severity of illness, age, sex, and mild or severe PVR were analyzed. We will also delete low-quality and/or medium-quality studies to check the robustness of the results.

#### 2.6.9 Sensitivity analysis

Sensitivity analysis can not only assess the stability and reliability of the conclusions of the Meta-analysis, but also assess whether the changes in the results are related to the impact of a single study. If the stability of the conclusion is poor, we can achieve the purpose of increasing stability by changing the analysis model, inclusion and exclusion criteria, or excluding a certain type of literature.

### 2.7 Data synthesis

The results of the study will be analyzed by RevMan 5.3 (Cochrane, London, United Kingdom) software provided by Cochrane collaborate on network. Whether a fixed effects model or a random effects model will be used depends on the results of the X^2^ test and I^2^ test for heterogeneity. If substantial statistical heterogeneity is not found, we will not pool the data but conduct a systematic narrative synthesis providing information to summarize and explain the characteristics and findings of the included studies.

### 2.8 Grading of quality of evidence

The Grading of Recommendations Assessment, Development, and Evaluation guidelines^[12]^ method will be applied to evaluate the quality of evidence of the pooled trials from5 aspects, included limitation of study design, inconsistency, indirectness, imprecision, and bias of publication. Additionally, the levels of evidence quality will be classified into 4 levels: high, moderate, low, and very low.

### 2.9 Ethics and dissemination

We will publish the system review results in peer-reviewed journals, disseminated in meetings or in peer-reviewed publications. Aggregated published data will be used to exclude data of individuals, so there is no need for obtaining the ethical approval or patients’ informed consent.

## 3 Discussion

*Hirudos*/Leechs are insect-like drugs, which are known as flesh-and-blood products, and are often used in clinical practice to promote blood circulation, remove blood stasis, relieve pain, relieve spasm, and extinguish wind.^[13]^ Constant studies have shown that *Hirudos*/Leechs contain 17 kinds of amino acids and proteins, including 8 kinds of essential amino acids for humans,^[14]^ as well as *Hirudin*, histamine, and heparin. In addition, the *Hirudos*/Leechs also contain small molecules such as glycolipids, carboxylic esters, and pteridines. In addition, there are 14 trace elements, such as Zn, Fe, Mn, Co, Se, Cr, Cu, and so on.^[15]^*Hirudos*/Leechs can play an anti-inflammatory, analgesic, anticoagulant, anti-fibrosis, anti-apoptosis, anti-tumor, and other such roles.^[16–20]^ Because *Hirudos*/Leechs have a strong role in promoting blood circulation and removing blood stasis, breaking the stasis will not damage new blood, and will not damage the Zhengqi, so *Hirudos*/Leechs can also be used as an important medicine to facilitate blood circulation and drive blood stasis in ophthalmology, and can therefore be widely used in a variety of ophthalmic diseases.

The most important cell type in PVR pathogenesis is the retinal pigment epithelial (RPE), which is deemed to dedifferentiate and migrate through a retinal break and then proliferate on the retinal layers and vitrea, resulting in formation of epiretinal membranes.^[21]^ The studies show *Hirudo* extract thereof and hirudin can inhibit the proliferation of RPE cells with P38 MAPK signaling pathways.^[22,23,24]^ However, the active ingredient of *Hirudos*/Leechs in the treatment of PVR has not been determined so far. But it can be inferred from some clinical studies that the greatful effectiveness of *Hirudos*/Leechs for PVR may be related to the main active ingredients such as *Hirudin*.

Therefore, the objective for this systematic review is to evaluate the efficacy and safety of *Hirudo* (Leech) or compound prescription containing *Hirudo* (Leech) for treating PVR. It is helpful to determine the potential value of *Hirudo* (Leech) therapies for PVR, as this can improve the quality of life of severe patients. This study cannot only provide a basis for releasing PVR treatment guidelines, but also promoting the application of TCM prescriptions so that more patients can benefit from them. However, this systematic review has several limitations. The quality of the study included is not up to standards and its methodology is not strict enough. The interventions of *Hirudo* (Leech) also vary from study to study. High heterogeneity may also exist due to inconsistencies in the included studies.

## Author contributions

**Conceptualization:** Yuanyuan Li.

**Data curation:** Qun Huang, Na Gao.

**Methodology:** Hui Huang, Ruxue Lei.

**Software:** Hui Huang, Weiwen Zou.

**Supervision:** Yuanyuan Li.

**Writing – original draft:** Hui Huang.

**Writing – review & editing:** Hui Huang, Yuanyuan Li.

## References

[1] Tseng W Cortez RT Ramirez G Prevalence and risk factors for proliferative vitreoretinopathy in eyes with rhegmatogenous retinal detachment but no previous vitreoretinal surgery. Am J Ophthalmol 2004;137:1105–15. 15183797 10.1016/j.ajo.2004.02.008

[2] Cardillo JA Stout JT LaBree L Post-traumatic proliferative vitreoretinopathy. The epidemiologic profile, onset, risk factors, and visual outcome. Ophthalmology 1997;104:1166–73. 9224471 10.1016/s0161-6420(97)30167-5

[3] Abrams GW Azen SP McCuen BW Vitrectomy with silicone oil or longacting gas in eyes with severe proliferative vitreoretinopathy: results of additional and long-term followup. Silicone study report 11. Arch Ophthalmol 1997;115:335–44. 9076205 10.1001/archopht.1997.01100150337005

[4] Pastor JC Rojas J Pastor-Idoate S Proliferative vitreoretinopathy: a new concept of disease pathogenesis and practical consequences. Prog Retin Eye Res 2016;51:125–55. 26209346 10.1016/j.preteyeres.2015.07.005

[5] Chen H Wang H An J Plumbagin induces RPE cell cycle arrest and apoptosis via p38 MARK and PI3K/AKT/mTOR signaling pathways in PVR. BMC Complement Altern Med 2018;18:89. 29534723 10.1186/s12906-018-2155-3 PMC5851073

[6] Heffer AM Proaño J Roztocil E The polyether ionophore salinomycin targets multiple cellular pathways to block proliferative vitreoretinopathy pathology. PLoS One 2019;14:e0222596. 31527897 10.1371/journal.pone.0222596 PMC6748436

[7] Wang XL Lei XQ Effect of Huyu Sanjie tablet on the hemorheologyof rabbit eyes with proliferative. vitreoretinopathy. Int J Ophthalmol (Guoji Yanke Zazhi) 2005;5:663–527.

[8] Huang YH Zeng JP Wong MF The inhibition effect of Huayu Sanjie tablet on the epiretinal membrane and platelet -derived growth factor with experimental proliferative vitroretinopath. Chin J Chin Ophthalmol 2007;17:270–3.

[9] Zheng YL Wang F Wang FJ Effect of Huayu Sanjie tablet on recovery of visual function of patients after retinal detachment surgery. J N Chin Med 2011;43:86–8.

[10] Moher D Shamseer L Clarke M Preferred Reporting Items for Systematic Review and Meta-Analysis Protocols (PRISMA-P) 2015 statement. Syst Rev 2015;4:1–9. 25554246 10.1186/2046-4053-4-1 PMC4320440

[11] Higgins J Green S 2011 Cochrane handbook for systematic reviews of interventions Version 5.1.0 [updated March 2011]. Available at: http://handbook-5-1.cochrane.org/.

[12] Balshem H Helfand M Schünemann HJ GRADE guidelines: 3. rating the quality of evidence. J Clin Epidemiol 2011;64:401–6. 21208779 10.1016/j.jclinepi.2010.07.015

[13] National Pharmacopoeia Editorial Board. The Pharmacopoeia of the P∗e∗o∗p∗l∗e's R∗e∗p∗u∗b∗l∗ic of China is one edition in 2020. Beijing: China Medical Science and Technology; 2020.

[14] Xu L Li X Zhang E The effect of leech extracts on endothelial cell coagulation-related factors and endothelial dysfuction-related molecules. Clin Exp Hypertens 2019;41:220–30. 29672166 10.1080/10641963.2018.1465076

[15] Li GQ L.I. Y.Y. L.I.T. Chemical constituents from Whitmania pigra. Tianjin J Tradit Chin Med 2018;35:703–5.

[16] Baranzini N De Vito A Orlandi VT Hirudo verbena antimicrobial role of RNASET2 protein during innate immune response in the medicinal leech. Front Immunol 2020;11:370. 32210967 10.3389/fimmu.2020.00370 PMC7068815

[17] Chandler J Cumpston M Thomas J Cochrane Handbook for Systematic Reviews of Interventions version 6.1 (updated September 2020). Cochrane, 2020. Available from www.training.cochrane.org/handbook. Accessed date: November 2020.

[18] Stange R Moser C Hopfenmueller W Randomised controlled trial with medical leeches for osteoarthritis of the knee. Complement Ther Med 2012;20:1–7. 22305242 10.1016/j.ctim.2011.10.006

[19] Sig AK Guney M Uskudar Guclu A Medicinal leech therapy-an overall perspective. Integr Med Res 2017;6:337–43. 29296560 10.1016/j.imr.2017.08.001 PMC5741396

[20] Ji Y Li L Wu MH Research progress in studies on antitumor mechanisms of leech. Chin J Inf Tradit Chin Med 2015;22:131–3.

[21] Pan ST Qin Y Zhou ZW Plumbagin induces G2/M arrest, apoptosis, and autophagy via p38 MAPK- and PI3K/Akt/mTOR-mediated pathways in human tongue squamous cell carcinoma cells. Drug Des Devel Ther 2015;9:1601–26. 10.2147/DDDT.S76057 PMC4365758 25834400

[22] Zheng YL Wang MF Sheng R ffect of hirudinoid extract on retinal pigment epithelium for proliferation mduced by thrombin. Chin J Chin Ophthalmol 2005;15:199–201.

[23] Bastiaans J van Meurs JC Mulder VC The role of thrombin in proliferative vitreoretinopathy. Invest Ophthalmol Vis Sci 2014;55:4659–66. 25015355 10.1167/iovs.14-14818

[24] Zheng YL Li Y Chen XL Influence of leech extract on signal transduction of human retinal pigment epithelir cell mediated by p38 mitogen activated protein kinase. Rec Adv Ophthalmol 2009;29:321–4.

